# Two Cases in Which Litholapaxy Was Impossible

**Published:** 1889-05

**Authors:** Edmund Andrews

**Affiliations:** Professor of Clinical Surgery, Chicago Medical College; No 6 Sixteenth Street


### TWO CASES IN WHICH LITHOLAPAXY WAS IMPOSSIBLE.

BY EDMUND ANDREWS, M. D., LL.D.,
PROFESSOR OF CLINICAL SURGERY, CHICAGO MEDICAL COLLEGE.

The operation of litholapaxy is applicable to almost all adult cases of vesical calculus, yet a few instances occur in which it cannot be performed, of which the following cases are examples:

*Case 1.*—This patient was aged 23 years, and somewhat broken in health by the cystitis caused by his calculus. The urine, however, was free from any signs of sugar or kidney casts, and showed only so much albumen as might come from the pus formed in the bladder. Bimanual examination showed no enlargement nor tenderness of the kidneys, so that in spite of the discovery of a very large stone in the bladder, and of the presence of a good deal of cystitis, he was not in a desperately bad constitutional condition. I prepared him for litholapaxy by washing out the bladder for two days with antiseptics, and giving quinine freely.

The rectum having been emptied, the patient was anæsthetized, and again sounded. The stone lay back close to the rectum, and the bladder was deformed by the presence of an inflammatory mass in front, pressing the anterior wall backward. After filling the bladder with warm carbolized water and introducing a lithotrite of large size, I found that the jaws had to be spread very widely to seize the stone, and when force was applied they slipped off without breaking it. Many repetitions all ended in the same way. The jaws would not hold, no matter on what side they seized the calculus. Litholapaxy was impossible, and I was compelled to desist and to perform supra-pubic lithotomy instead. The pubis being shaved and disinfected, I introduced into the bladder the French instrument called the *sonde à dard*, which is a long and large canula, having the shape of a silver catheter, but carrying in its interior a curved steel dart with a groove on its anterior surface. The object is to insert the instrument enclosing the dart and press its tip against the front of the bladder close to the top of the pubis so that it can be felt with the finger in the bottom of the primary incision, and then to protrude the point of the dart through the bladder into the external wound, and finally enlarge the puncture by using the dart as a grooved director, and sliding a bistoury along it into the bladder.

The instrument was a total failure in this case. The chronic inflammation of the viscus had extended through the front wall of the bladder and developed a thick mass of indurated cellular tissue through which the sound could not be felt. Even the sharp dart could not be made to pierce it. The *sonde à dard* failed as completely as the lithotrite had done, and the incision was therefore carried down in the ordinary way with the scalpel alone, and the cavity with the stone in it was at last reached well down against the rectum, after a dissection of alarming depth. The stone was then extracted without very great difficulty, though I was compelled to enlarge the wound considerably, on account of the great size of the calculus. It measured over three inches in diameter. On trying the lithotrite upon it outside the body, the reason of the failure of that instrument to bite through it was obvious. The jaws of even the largest lithotrite are too short to grasp the center of a stone three inches in diameter. The tips of the beaks catch feebly on the sloping sides, and instantly slip off without breaking the stone.

The wound was dressed antiseptically and without sutures. It healed pretty rapidly, considering its large size, and the patient recovered without any dangerous symptoms.

*Case 2.*—A boy 17 years of age had a stone of about an inch and a quarter in diameter, which proved to be composed of calcium oxalate. The usual preparations were made for litholopaxy, and the patient placed on the table and etherized. On seizing the stone it was found excessively hard, and resisted the utmost strength of my hands in trying to break it. As the instrument was made by one of the very best Eastern makers, and I had tested it on pieces of hard brick when I bought it, I had great confidence in its strength. I therefore requested my assistant to grasp the handle of the female blade and hold it firmly with both hands, while I turned with the strength of both my own hands on the male blade, thus doubling the power. At first we effected nothing, but a little later, as we put forth our whole strength, there came a sound of a smothered metallic clink from within. I immediately loosened the grip and shook the stone loose from the jaws, and then discovered that the blades were held apart, and for some time could not be closed. However, after repeated trials the jaws closed together and I drew the instrument out. The female blade was found cracked half across, about two inches from the inner end, and a slight spring of the broken edge inward was what caught the male blade and prevented the closure. It was an awkward situation, for the instrument could not be drawn out with the jaws open, and had I been totally unable to close them, I should have been obliged to open the bladder widely through the perineum, pry the jaws downward through the wound and send for a mechanic to file off the shank, as in no other way couldthe instrument be gotten out.

My mental equanimity, therefore, was greatly promoted by the closure of the jaws, and I set to work very cheerfully to perform lateral lithotomy in the usual manner, removing without difficulty the hardest stone I ever met. I at first fancied I should find some metallic object in it as a nucleus, but on sawing it open, I found nothing but the usual blackish oxalate to the center.

The patient recovered without the slightest unfavorable symptom.

The moral of the tale is this, that however well an instrument of this sort may be constructed, it is not safe to apply the force of two strong men to the twisting of its handles.

I think, however, that a special lithotrite could be constructed for these rare cases, with a larger shank, and with jaws a little deeper from back to front, which would break the hardest calculus, unless it were accreted on a piece of iron or steel for a nucleus. It would also be necessary to increase the power, either by giving the thread of the screw a lower pitch or increasing the diameter of the handles.

No 6 Sixteenth Street.

